# Evaluation of molecular descriptors for antitumor drugs with respect to noncovalent binding to DNA and antiproliferative activity

**DOI:** 10.1186/1471-2210-9-11

**Published:** 2009-09-16

**Authors:** José Portugal

**Affiliations:** 1 Instituto de Biología Molecular de Barcelona, CSIC, Parc Cientific de Barcelona, Baldiri Reixac, 10, E-08028 Barcelona, Spain

## Abstract

**Background:**

Small molecules that bind reversibly to DNA are among the antitumor drugs currently used in chemotherapy. In the pursuit of a more rational approach to cancer chemotherapy based upon these molecules, it is necessary to exploit the interdependency between DNA-binding affinity, sequence selectivity and cytotoxicity. For drugs binding noncovalently to DNA, it is worth exploring whether molecular descriptors, such as their molecular weight or the number of potential hydrogen acceptors/donors, can account for their DNA-binding affinity and cytotoxicity.

**Results:**

Fifteen antitumor agents, which are in clinical use or being evaluated as part of the National Cancer Institute's drug screening effort, were analyzed *in silico *to assess the contribution of various molecular descriptors to their DNA-binding affinity, and the capacity of the descriptors and DNA-binding constants for predicting cell cytotoxicity. Equations to predict drug-DNA binding constants and growth-inhibitory concentrations were obtained by multiple regression following rigorous statistical procedures.

**Conclusion:**

For drugs binding reversibly to DNA, both their strength of binding and their cytoxicity are fairly predicted from molecular descriptors by using multiple regression methods. The equations derived may be useful for rational drug design. The results obtained agree with that compounds more active across the National Cancer Institute's 60-cell line data set tend to have common structural features.

## Background

DNA-binding molecules represent a valuable portion of the clinically useful antitumor drugs [1,2]. Most of the drugs than bind noncovalently to DNA, such as actinomycin D and several anthracyclines [1,3], interact selectively with the nucleic acid along the minor groove or by intercalation. The binding mode depends on structural features of these molecules and on the DNA sequences they recognize [4-6]. The strength of reversible binding to DNA can be quantified for any drug by means of the equilibrium binding constant (*Keq*). In the determination of the binding constant, the primary results obtained from whatever the technique used and the analysis of the data are not straightforward as there is no a single protocol that might be applied to every 'binding problem' [7]. Therefore, DNA-binding data, which may be used to correlate noncovalent drug-DNA interactions with cytotoxicity data, should be regarded as 'approximate values' compared to the more accurate measurements that are available on some physicochemical molecular descriptors for these molecules, such as molecular weight, hydrophobicity or the number of hydrogen bond donors.

The analysis of antitumor drugs based on an evaluation of cytotoxicity data may provide new insights into their mechanism of action [2,8]. The 60 human cancer cell lines used in the screening of compounds at the National Cancer Institute (NCI) provide basically the GI_50 _(50% growth-inhibitory concentration) as an index of cytotoxicity or cytostasis. The NCI cell line data set is a publicly available database that contains cellular assay screening data for over 40000 compounds tested in 60 human tumor cell lines (referred to hereafter as the NCI-60 cell lines). The database also contains microarray gene expression data, thus providing an excellent information resource particularly for the analysis of links between chemical, biological, and genomic information [2,8-11].

While it is worth characterizing cancer cells to predict chemosensitivity to any particular drug [12], and to link changes in gene expression to cytotoxicity [8,13-15] it is also of utmost importance a deeper understanding of the mechanism of action of drugs, which includes the dissection of forces driving their noncovalent binding to DNA, and to use this information to help in the development of new anti-cancer agents with higher activity [9,16,17].

This paper presents the analysis of the relationship between various physicochemical descriptors for drugs, and uses these descriptors to predict both the strength of noncovalent binding to DNA and the biological activity. Although there are previous studies considering that large and more complex molecules are more potent antitumor agents [9], there is no clear association between biological potency and the strength of reversible binding to DNA, while there are some examples illustrating that changes in DNA binding among structurally related molecules can be accompanied by abrupt changes in biological activity [18]. The strategy followed here utilizes DNA-binding constants taken from the ample, and sometimes contradictory, bibliography on DNA-binding drugs. The *Keq *values, shown as logarithmic-transformed values *logKeq *in Table 1, were selected following the same criteria used elsewhere [5] to analyze the signature for drug-DNA binding modes.

**Table 1:** Molecular descriptors for noncovalent DNA-binding drugs^a^.

	**NSC number**	**DNA binding**	**Mw**	**XlogP**	**HbD**	**HbA**	**PSA**	**Complexity**	**log *Keq***	**Lipinski**	**GI_50_**
Actinomycin D	3053	Intercalation	1255	1.6	5	18	356	3030	5.38	2	8.7
Bleomycin	125066	Intercalation	1416	-1.9	20	30	627	2580	5.57	1	5.9
Chartreusin	5159	Intercalation	641	2.6	5	14	200	1150	5.45	2	5.7
Chromomycin	58514	minor-groove	1185	-0.6	8	26	359	2480	4.41	1	8.3
Daunorubicin	82151	intercalation	528	0.1	5	11	186	960	6.65	2	7.1
Distamycin A	82150	minor-groove	482	0.2	6	9	181	825	6.89	3	4.1
Doxorubicin	123127	intercalation	544	-0.5	6	12	206	977	6.30	1	7.2
Echinomycin	526417	bis-intercalation	1101	2.5	4	16	302	2200	5.52	2	8.1
Elsamicin A	369327	intercalation	654	2.9	5	14	206	1210	6.54	2	7.5
Epirubicin	256942	intercalation	544	-0.5	6	12	206	977	6.57	2	6.7
Ethidium	268986	intercalation	394	4.3	2	3	56	419	4.90	4	5.5
m-AMSA	249992	intercalation	394	3.8	2	6	80	601	4.30	4	6.2
Mitoxantrone	301739	intercalation	445	-3.1	8	10	163	571	6.78	3	7.2
Mithramycin A	24559	minor-groove	1085	-0.4	11	24	358	1940	5.08	1	7.9
Netropsin	3067	minor-groove	431	-1.7	7	10	211	723	6.40	2	4.0

^a ^Mw: Molecular weight, XlogP: partition coefficient that measures the differential solubility of a compound in two solvents, HbD: number of hydrogen bond donors in the structure, HbA: number of hydrogen bond acceptors, PSA: polar surface area (in Å^2^). Complexity: a rough estimate of how complicated a structure is. Both the elements contained and the displayed structural features including symmetry are considered. log *Keq*: logarithmic-transformed equilibrium binding constant for a drug-DNA complex (*Keq *in M^-1^). Lipinski: Lipinski's score, the rule-of-five value used to measure bioavailability. GI_50 _is used in place of -log(GI_50_), the negative logarithm of the average drug concentration that inhibits cell growth in the NCI-60 cell lines (August 2008 data) as a measure of cytotoxicity or cytostasis.

It was feasible to achieve the required robustness for an *in silico *study based on a relatively small sample population by using different and, to some extent, complementary approaches under a careful statistical control. It was, therefore, possible to derive equations to predict the strength of binding to DNA and the biological activity (cytotoxicity) by multiple regression methods using a combination of structure-based molecular descriptors and some other physicochemical descriptors as predicting variables. Moreover, factor analysis was used to uncover the latent structure (dimensions) of the molecular descriptors. Principal component analysis is exposed as a valuable tool for predicting of redundancy of descriptive elements in drug design. Both the strength of noncovalent binding to DNA and cytotoxicity might be predicted, even though not perfectly, from molecular descriptors.

## Methods

### Noncovalent DNA-binding drugs: A database of molecular descriptors and growth inhibition response

The antitumor drugs used in the present analysis were selected by two complementary criteria. First, they are drugs binding noncovalently to DNA whose binding affinity, measured as the equilibrium binding constant *Keq*, as well as their mechanism of binding--intercalation or minor-groove binding--have been fully established previously. Second, their cytotoxicity assays using the NCI-60 cell lines are publicly available through the Developmental Therapeutics Program NCI/NIH database at:  (cancer screen data, August 2008). For the sake of convenience, GI_50_, the 50% cancer cell growth inhibition concentration for any particular cell line, a measure of cytotoxicity or cytostasis, is used to indicate the -log(GI)_50 _provided by the database. The GI_50 _measures shown in Table 1 are the arithmetic mean of the GI_50 _measurements in these 60 cell lines.

Common molecular descriptors for all the drugs were retrieved from the PubChem compound web site: . Lipinski's scores were retrieved from the ChemDB (NIAID) database . The drug-DNA equilibrium binding constants (*Keq*) were obtained from a survey of the vast, and, sometimes, contradictory information found in the bibliography (see Results). The equilibrium binding constants entered in Table 1 correspond almost exclusively to those acquired under similar experimental conditions: 20-25°C, pH 7 and 150-200 mM NaCl, in accordance with an uniform criteria used elsewhere to establish a thermodynamic signature for the drug-DNA mode of interaction [5]. Throughout this paper the logarithmic-transformed values (log *Keq*) are used for the convenience of normalization of the data.

**Figure 1:**
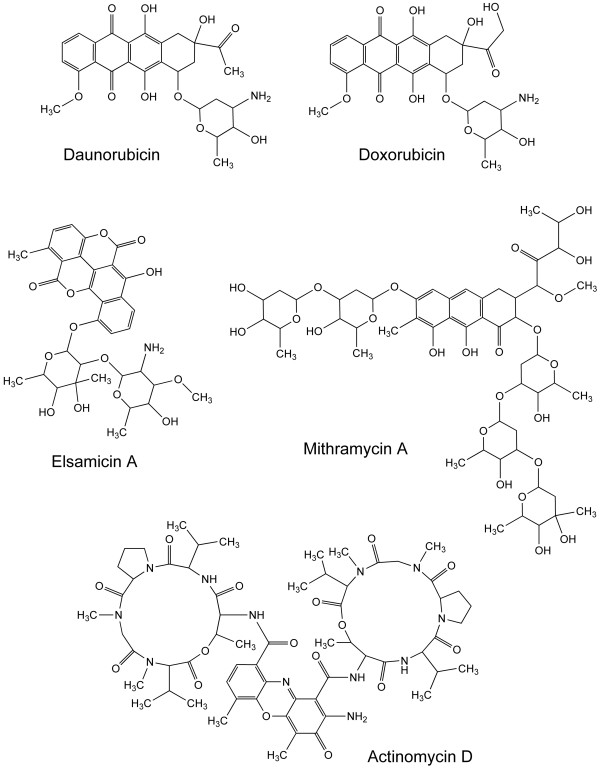
**Molecular formulae of six of the noncovalent DNA-binding drugs used in the present study**. The drugs displayed are characterized by their high activity in the NCI's tumor screening panel.

Fifteen antitumor drugs (Table 1 and Fig. 1), most of them in clinical use, were deemed to achieve the criteria required to enter the present study.

### Correlation and Multiple Regression

Most statistical calculations were carried out using the SPSS v.13.0.1 package (SPSS Inc., Chicago, IL). The normality (normal distribution) of the data was analyzed by the Shapiro-Wilk test, which is especially suitable for analyzing samples containing a small number of variables [19]. Both the Pearson and Spearman's ρ correlation coefficients (one tailed tests) have been calculated among the different molecular descriptors. Pearson coefficients require assuming a normal distribution of the sample, while the Spearman's ρ is a non-parametric measure. The actual *p *values are usually indicated for the different statistic analyses.

Multiple regression was used to predict *Keq *from the molecular descriptors described in the legend to Table 1. Multiple linear regression benefits from a well-developed mathematical framework that yields unique solutions and exact confidence intervals for regression coefficients [20]. As the first choice, the regression procedure followed was *stepwise selection *(criteria used: probability-of-F-to-enter ≤ 0.050, probability-of-F-to-remove ≥ 0.100), although other available methods as *entry *and *backward *were also used to reach the better, statistically significant, estimation of *logKeq*. Multiple regression was also used to predict GI_50_, using the same molecular descriptors plus Lipinski's scores and *logKe*q values as additional variables.

The model output, generated by the SPSS software package, provides several parameters that were used to check the reliability of the analysis, such as the correlation coefficient (r), r^2 ^that indicates the percentage of the variation in *logKeq *(or GI_50_) that can be explained by the regression, and the adjusted r^2 ^(AdR^2^) that is the r^2^corrected for the number of predictors.

AdR^2 ^was determined by the following equation:



in which n = number of cases (drugs) and k = number of variables (descriptors)

AdR^2 ^was computed to avoid an overestimation in predicting r^2 ^due to the few cases (15 drugs) tested relative to the number of variable (6 molecular descriptors)--up to 8 variables when Lipinski's rule scores and *logKeq *values were introduced in the analyses of GI_50_, see results--.

### Multicollinearity

Multicollinearity, also known as collinearity, arises when a high degree of correlation (either positive or negative) exists between two or more independent variables. Because multicollinearity means redundancy in the molecular descriptors to predict *logKeq *or GI_50_, its presence was detected by determining the *variance inflation factor *(VIF), which can be computed from individual r^2^--distinct from the overall r^2 ^of the model--using the following equation:



When variables are collinear VIF is higher than 1. A VIF of 4 and above was used to detect a multicollinearity problem (corresponding to r^2 ^values greater than 0.75) and used to eliminate the corresponding molecular descriptors from predictive equations.

### Hierarchical cluster analysis of molecular descriptors and DNA-binding drugs

Cluster analysis was performed with the SPSS software, using a single linkage (agglomeration) algorithm method and Pearson correlation coefficients. Dendrograms showing hierarchical clustering are presented in terms of similarities between cases (DNA-binding drugs) or variables (molecular descriptors).

### Principal Component Analysis

Principal component analysis was undertaken as a way of identifying patterns in data, and expressing them in such a way as to highlight their similarities and differences. Principal component analysis extracted factors on the basis of the correlation between the six molecular descriptors in Table 1 and *logKeq*. For GI_50 _analysis, *logKeq *and Lipinski's scores (Table 1) were used as additional predictor variables.

Principal component with *eigenvalues *of 2 were retained and orthogonally rotated using the *Varimax *method -- the point was to minimize the complexity of the component while ensuring that the molecular descriptors were uncorrelated--. Descriptors with loadings of ≥ 0·25 or ≤ -0·25 were considered as significant contributors. Factor scores were saved for each principal component, and used to obtain graphic representations of drug's scores, on 2D principal component plots.

## Results

### Drugs that bind reversibly to DNA can be characterized by common molecular descriptors, as well as by their DNA-binding constant

Table 1 shows six common molecular descriptors, XlogP, molecular weight (Mw), number of potential hydrogen bond acceptors (HbA), potential hydrogen bond donors (HbD), polar surface area (PSA) and complexity. The complexity rating of a compound is the rough estimate of how complicated a structure is, seen from both the point of view of the elements contained and the displayed structural features including symmetry. The complexity values, computed using the Berz/Hendrickson/Ihlenfeldt formula, were retrieved from the PubChem compound web site: . Table 1 also presents the equilibrium DNA binding constant for fifteen drugs, retrieved from the bibliography [4,5,16,21-31] (the values of *Keq *have been transformed to *logKeq *in the sake of data normalization). Table 1 also displays the values of the Lipinski's score (a measure of bioavailability [32]), and the GI_50_, which is a measure of the cytotoxicity or cytostasis induced by the different drugs [2]--it corresponds to the negative logarithm of the drug concentration that inhibits cell growth--. Table 1 contains molecules binding to DNA reversibly [4], yet some of them are also classified as topoisomerase II poisons, or they are known to be involved in reactions leading to DNA cleavage. The set of DNA-binding drugs encloses intercalators, the bis-intercalator echinomycin, and some minor-groove binders.

The number of variables (molecular descriptors) and drugs that accomplished the prerequisites to enter the study was rather small; thereby normality of the sample distribution could be compromised. According to the Shapiro-Wilk normality test (Table 2), only Mw, and HbD departed significantly from normal distribution (*p *> 0.01). Furthermore, other precautions needed in a statistical analysis of small populations were undertaken, and they are indicated in the relevant place in the text.

**Table 2:** Results of the Shapiro-Wilk normality test^(a)^

	**W-Statistic**	**df**	***P***
MW	0.827	15	0.008
XlogP	0.951	15	0.542
HbD	0.767	15	0.001
HbA	0.933	15	0.298
PSA	0.862	15	0.026
Complexity	0.873	15	0.038
logKeq	0.915	15	0.163
Lipinski	0.847	15	0.016
GI_50_	0.960	15	0.697

^(a) ^Molecular descriptors were considered to pass the normality test if *p *> 0.01

The fifteen drugs described in Table 1 were tested as three subsets aimed at helping to parse the potential of molecular descriptors for predicting *logKeq *values. The first set contemplates all the DNA-binding drugs, while the other sets correspond to the intercalators (bis-intercalating echinomycin was not considered a member of this subset), and to DNA-binding drugs that had been organized into self-organizing maps (SOM) as belonging to the 'M-region' (so, named here as 'M-region' compounds) [9]. 'M region' compounds possess an outstanding cytotoxic, or cytostatic, activity (high GI_50 _values) and relatively larger PSA and Mw compared to other antitumor drugs analyzed in the NCI-60 cell lines [9]. Drugs belonging to this SOM region were obtained online by using the 3D Mind tools at: . They are actinomycin D, chromomycin, daunorubicin, doxorubicin, echinomycin, elsamicin A, mithramycin A and mitoxantrone.

Correlations between the drug binding constant (*logKeq*) and the different molecular descriptors were calculated by using two different coefficients: the Pearson correlation coefficient, which is a parametric statistic, and the non-parametric Spearman's ρ correlation coefficient, which might be more reliable for the molecular descriptors that did not show a normal distribution (Table 3). The Pearson and Spearman's ρ correlations calculated between each molecule descriptors plus *logKeq *and GI_50 _values are shown, together with the significance levels, in Additional Files 1 and 2, respectively.

**Table 3:** Calculated Pearson and Spearman's rgcorrelation coefficients between each molecular descriptor and *log Keq*.

		**All Drugs**				**Intercalators**				**'M-region'**		
	**Pearson**	**p**	**Spearman's**	**p^a^**	**Pearson**	**p**	**Spearman's**	**p**	**Pearson**	**p**	**Spearman's**	**P**
Mw	-0.396	7.22 × 10^-2^	-0.177	2.64 × 10^-1^	-0.096	3.96 × 10^-1^	0.073	4.20 × 10^-1^	-0.909	8.72 × 10^-4^	-0.857	3.27 × 10^-3^
XlogP	-0.461	4.20 × 10^-2^	-0.388	7.66 × 10^-2^	-0.662	1.85 × 10^-2^	-0.699	1.20 × 10^-2^	-0.118	3.91 × 10^-1^	0.000	5.00 × 10^-1^
HbD	0.047	4.34 × 10^-1^	0.252	1.83 × 10^-1^	0.189	3.01 × 10^-1^	0.636	2.40 × 10^-2^	-0.368	1.85 × 10^-1^	-0.258	2.69 × 10^-1^
HbA	-0.272	1.63 × 10^-1^	-0.244	1.91 × 10^-1^	0.143	3.50 × 10^-1^	0.049	4.47 × 10^-1^	-0.955	1.11 × 10^-4^	-0.976	1.66 × 10^-5^
PSA	-0.151	2.96 × 10^-1^	-0.211	2.25 × 10^-1^	0.079	4.10 × 10^-1^	0.141	3.50 × 10^-1^	-0.950	1.47 × 10^-4^	-0.994	1.00 × 10^-6^
Complexity	-0.373	8.56 × 10^-2^	-0.252	1.82 × 10^-1^	-0.088	4.00 × 10^-1^	-0.067	4.30 × 10^-1^	-0.844	4.18 × 10^-3^	-0.857	3.27 × 10^-3^

^a ^Significance level (actual *p *values)

Significant correlations between *logKeq *and several molecular descriptors were established according to both the Pearson and Spearman's ρ coefficients (Table 3)--see also Additional Files 1 and 2--. These results indicated that it was reliable to use the more robust parametric tests throughout the present study despite the, from a statistical point of view, small size of the sample analyzed--when corrections for small sample were available they were thoroughly used--, or the departure from normality of a few parameters. In general, there was a clear correspondence between the correlation values obtained by using either coefficient, yet the actual *p *values differed (Table 3). The molecular descriptors that correlated better, either positively or negatively, with *logKeq *were not the same when the fifteen drugs were evaluated together, or the correlations were calculated for 'intercalators' and 'M-region' compounds respectively. According to the Pearson correlation coefficients, when all the drugs were analyzed together there was a negative correlation with XlogP (*p *< 0.05) but also with Mw and complexity, although with *p *< 0.1 (Table 3). According to the Spearman's ρ, only XlogP correlated negatively with *logKeq *(*p *< 0.1). For the DNA intercalators, both coefficients showed a negative correlation between *logKeq *and XlogP; while the Spearman's ρ also indicated a significant positive correlation with HbD (Table 3). The number of molecular descriptors that correlated significantly with *logKeq *was clearly higher when the more active 'M region' compounds were studied (Table 3). Both coefficients revealed significant correlations between *logKeq *and four molecular descriptors: Mw, HbA, PSA and complexity (*p *< 0.01). Nevertheless, some of these descriptors were also highly correlated among them, and therefore there are grounds for considering that any intention of deriving equations to predict *logKeq *for any DNA-binding molecule based upon a combination of these molecular descriptors should not disregard that they can contain redundant information. The only molecular descriptor that seemed to be relatively independent of the other descriptors was XlogP, tentatively because it is a molecular descriptor for hydrophobicity almost independent of the size of the molecules.

### Drug-DNA binding constants might be predicted from a set of molecular descriptors

Multiple linear regression calculations were used to derive equations to predict *logKeq *for drugs binding reversibly to DNA by using the set of molecular descriptors shown in Table 1. If these equations are to be used in drug analysis, a multiple regression approach would require to consider the presence of redundant information among the molecular descriptors, because several molecular descriptors showed a fair correlation with *logKeq *(Table 3), thus redundancy (multicollinearity, see Methods) had to be avoided. The number of cases (i. e., noncovalent DNA-binding drugs) should substantially exceed the number of predictor variables to be used in a multiple regression analysis (one rule of thumb is to have at least five times more cases as predictor variables). At first glance, this is a condition that seems impossible to meet here. Nevertheless, since redundancy may be eliminated, as shown below, the number of variables (molecular descriptors) was reduced; thereby the variables to cases ratio became acceptable.

**Figure 2:**
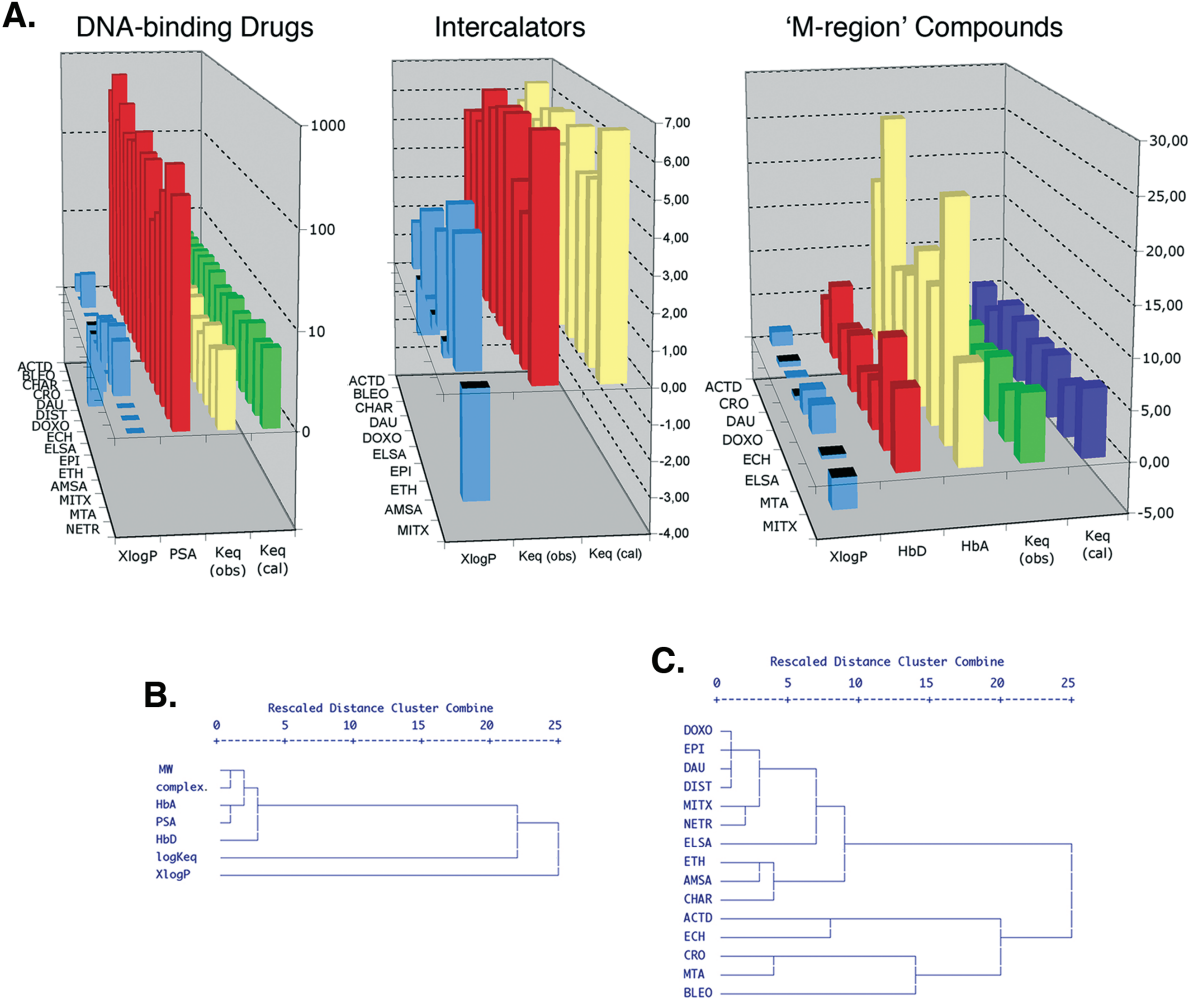
**Common molecular descriptors and noncovalent binding to DNA**. **(A) **Predicted *logKeq *values obtained by multiple regression analysis using molecular descriptors (Keq (cal)) are plotted together with experimentally calculated values (Keq (cal)) for the complete set of drugs, intercalators and 'M region' compounds respectively. Equations used to calculate *logKeq *are shown in Table 4. ACT (actinomycin D), BLEO (bleomycin), CHAR (chartreusin), CRO (chromomycin), DAU (daunorubicin), DIST (distamycin), DOXO (doxorubicin), ECH (echinomycin), ELSA (elsamicin A), EPI (epirubicin), ETH (ethidium), AMSA (m-AMSA), MTA (mithramycin A), NETR (netropsin). **(B) **Dendogram showing average linkage hierarchical clustering of six molecular descriptors for noncovalent DNA-binding drugs, based on the Pearson correlation coefficients. Descriptors with higher similarity are clustered together. **(C) **Hierarchical clustering applied to the 15 drugs binding reversibly to DNA (Table 1) on the basis of their proximities. Connecting lines further to the right indicate more distance between clusters of either molecular descriptors (B) or drugs (C).

Table 4 presents the equations obtained by multiple linear regression that predict *logKeq *by using some of the molecular descriptors as variables. All the equations were derived following two criteria. First, they contained predictors in absence of multicollinearity, thus avoiding redundancy owing to the high correlation between some of the molecular descriptors. The VIP (variance inflation factor) was used to eliminate any multicollinearity--see Methods and Additional File 3--, which explains the statistic details. Second, they were statistically significant according to an ANOVA test (Table 4, and Additional File 3). When the 15 drugs were analyzed together, *logKeq *values were better predicted by XlogP (the correlation was negative), yet the polar surface area (PSA) could also participate (*p *< 0.1; Table 4 and Fig. 2A). The prediction of *logKeq *using these molecular descriptors was low 36% according to the r^2 ^values, corresponding to about 26% when an adjusted r^2^, which accounts for the small size of the sample, was used (AdR^2 ^in Table 4) and barely significant (Table 4). Much better predictions of *logKeq *were obtained when drugs were considered as the subsets 'intercalators' and 'M-region compounds' described above. For intercalators, about 44% (37% using the adjusted parameter) of the *logKeq *values were explained by XlogP (*p *< 0.05; Table 4 and Fig. 2A). The better prediction of *logKeq *was obtained for the drugs included in the 'M-region', for which two equations were derived from multiple correlation models (ANOVA test, p ≤ 0.002), with a prediction reaching more than 90% (almost the same value was observed when the AdR^2 ^was considered, Table 4). It is noteworthy that for this particular set of drugs XlogP contribution appeared to be less important than the HbA and HbD values. This observation is in keeping with the ranks of molecular descriptors and mean GI_50 _used to define the 'M-region' in SOM analysis [9] (see also Fig. 2A).

**Table 4:** Equations used to predict *logKeq *values for DNA-binding drugs^a^.

	**Predictive equation**	**r**	**p**	**AdR^2^**
All drugs	**log *Keq *= -0.255(± 0.100) XlogP - 0.003(± 0.002)PSA + 6.603(± 0.465)**	0.603	6.6 × 10^-2^	0.258
All drugs	**log *Keq *= -0.181(± 0.097)XlogP + 5.865(± 0.215)**	0.461	8.4 × 10^-2^	0.152
Intercalators	**log *Keq *= -0.225(± 0.090)XlogP + 6.054(± 0.229)**	0.662	3.7 × 10^-2^	0.368
'M-region'	**log *Keq *= 0.128(± 0.067) XlogP - 0.178(± 0.020)HbA + 0.173(± 0.066)HbD + 7.577(± 0.268)**	0.984	2.0 × 10^-3^	0.944
'M-region'	**log *Keq *= -0.138(± 0.190)HbA + 8.090(± 0.303)**	0.955	2.2 × 10^-4^	0.897

The predictive equations are presented for the three sets of drugs analyzed (All DNA-binding drugs, Intercalators and 'M-region' compounds) described in the main text^b^.

^a^Obtained by multiple regression analysis, in which molecular descriptors showing multicollinearity were discarded (see the main text for details). The predictive equations displayed are those statistically "more significant" for each set of predictors (actual *p *values, ANOVA test, are shown in the Table), *r *is the correlation coefficient of the linear fit, *AdR*^2 ^is the fraction of the variance in *logKeq *that is explained (predicted) by the model, corrected for the number the variables in the model, as described in Methods.

^b^The cases (drugs) used in the calculations for each set were 15, 10 and 8 respectively.

### Hierarchical cluster analysis builds groups of molecular descriptors and DNA-binding drugs based on their similarity

A hierarchical clustering analysis of the different molecular descriptors based on the Pearson correlation coefficient was used to build groups of molecular descriptors showing close physicochemical properties. The dendrograms shown in Fig. 2B indicated that among the molecular descriptors there were only two independent predictors: XlogP (a round measure of lipophilicity or hydrophobicity) and molecular size, which clustered at larger distances. One of the clusters revealed the proximity among the variables that appear to depend on the molecule size, such as Mw or PSA, while the other two parameters XlogP and *logKeq *only clustered with them at large distances. This clustering visually showed that the different parameters were highly correlated, the exception being XlogP (Fig. 2B). These results were consistent with previous reports that used a larger data set that included drugs with multiple mechanisms of action [9]. This coincidence, which occurred regardless of the sample size, may be considered as an indirect proof of the robustness of the present approach using a smaller sample population.

Moreover, hierarchical analysis was used to classify the different drugs into relatively homogeneous groups within themselves and heterogeneous between each other, on the basis of the Pearson correlations between molecular descriptors (Fig. 2C). Dendrograms, showing the relationship among the six molecular descriptors used to predict *logKeq *for all the DNA-binding drugs are shown in Fig. 2C, with connection lines further to the right indicating more distance between drugs and clusters. This hierarchal classification grouped together molecules with similar structures, such as the anthracyclines doxorubicin, daunorubicin and epirubicin, while the more complex actinomycin D and echinomycin were also brought together in a different cluster, the latter clustering next to other large molecules, such as bleomycin and the structurally related aureolic acid antibiotics mithramycin A and chromomycin. The minor-groove binder distamycin clustered with the anthracyclines (Fig. 2C), in keeping with that all these molecules showed similar values in several parameters, which included *logKeq *and complexity (Table 1).

### Principal component analysis discovers and summarizes patterns of intercorrelation among molecular descriptors

The presence of multicollinearity in the multiple regression analysis raised a question on whether some of the parameters used in the analysis of drug's activity endure redundant information that may be reduced to a few key molecular descriptors conveying all the structural information required for drug design. A way to identify underlying variables (factors) is provided by principal component analysis, see Methods. The variables (molecular descriptors) for the fifteen noncovalent DNA-binding drugs were analyzed, using three separate computations corresponding to the three subsets of drugs described above.

**Figure 3:**
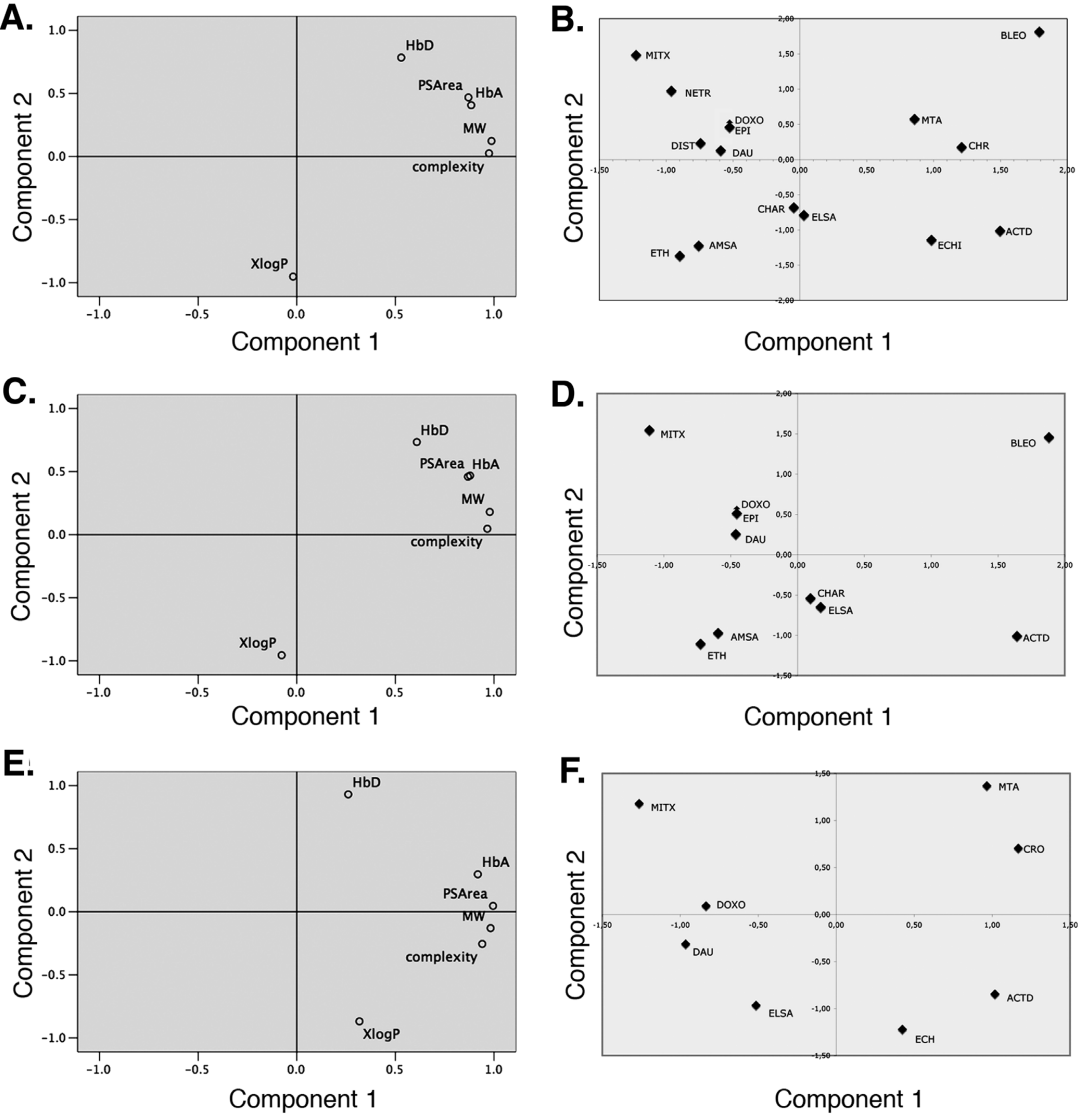
**Principal component analysis of molecular descriptors for noncovalent DNA-binding drugs**. Two-dimensional representation of loading values and factor scores on principal components 1 and 2 are shown in a rotated space. Component 1 may be labeled "molecular size" while component 2 would be "hydrophilicity-hydrophobicity", with XlogP clearly loading in the hydrophobic part of the axis. Loadings are displayed side-by-side with a representation of the factor scores for the different drugs on the two principal components. Graphical representations correspond to the analysis of all fifteen noncovalent DNA-binding drugs **(A) (B)**, intercalators **(C) (D)**, and the 'M-region' compounds **(E) (F)**, respectively.

Fig. 3 shows the results of a principal component analysis, in which the molecular descriptors were plotted on the first two components. In principal component analysis the measure of the percent of variance in a given variable explained by all the factors is known as communality. Communalities corresponding to the different principal component analysis are shown as supplementary data (Additional File 4), which also presents other statistic details of the principal component analysis. For all drugs, the extracted communalities were, in general, over 90%, which means that most of the percent of variance in a given molecular descriptor was explained by the factors (components) extracted. When all the drugs were considered together the first principal component explained 74.22% of total variance, while 20.13% was explained by the second component (Fig. 3A). In summary, the two principal component models were enough to accurately describe the data, since they explained 94.35% of the variance. Component 1 can be considered to reflect the "molecular size" while component 2 would represent a "hydrophilicity-hydrophobicity" axis, with XlogP clearly loading in the hydrophobic part of it. Graphic representation of the scores obtained by principal component analysis offered a direct visual identification of some common features of the drugs (Fig. 3B). For example, structurally-related drugs, such as the anthracyclines, clustered together in the same regions of the plot. Besides, the larger molecules also clustered nearby (echinomycin, actinomycin D), or also the smaller ones (ethidium and m-AMSA). There were some differences in the exact location (score) on the graphic representation depending on the subset of data used: all drugs (Fig. 3B), intercalators (Fig. 3D) or 'M-region' compounds (Fig. 3F)).

The larger bleomycin, mithramycin A and chromomycin (Table 1) were located in a region corresponding to the positive values of the first component, on account of their "big size" compared to the relatively smaller doxorubicin and daunorubicin (Fig. 3B). Figs. 3C and 3D show the principal component analysis for the set of DNA-intercalating drugs, for which the principal component explained 77.74% of the variance, and 17.35% was explained by the second component, with a total 95.09% of variance explained by both factors. Principal component for the 'M-region' compounds (Figs. 3E and 3F) were 64.11% and 29.87% respectively, with a 93.98% of the total variance explained. The number of potential hydrogen donors (HbD) had a loading value much closer to the hydrophilic factor in this component, opposite to XlogP loading (Fig. 3E). An elevated relevance of the number of potential hydrogen bonding in drugs belonging to the 'M-region' subset was in concordance with the importance given to them as a source of DNA-binding specificity [4,5].

### DNA-binding constants and a combination of molecular descriptors might be used to estimate the cytotoxicity (GI_50 _values) of drugs binding noncovalently to DNA

After obtaining equations that predict *logKeq *from the different molecular descriptors, we should consider whether a correspondence exists between the strength of noncovalent binding to DNA and the cytotoxicity or cytostasis measured as the GI_50 _values. At this point, the Lipinski's scores [32] (Table 1), an additional descriptor of biological activity for drugs (also known as the rule-of-five), was added for the following calculations.

For the complete set of DNA binding drugs a small negative and nonsignificant correlation was found between GI_50 _and *logKeq *(Pearson correlation coefficient: -0.277 (*p *= 3.17 × 10^-1^)), which for the more potent 'M-region' compounds was -0.822 (*p *= 1.51 × 10^-1^)). However, the correlation was positive, yet barely significant, when the subset of intercalators was considered (0.413; *p *= 1.17 × 10^-1^). Although, all these correlations were within the limits of being of random occurrence, it is noteworthy that cytotoxicity was not positively related to *logKeq*, except for the intercalating agents. Indeed, the 'M-region' encompasses several intercalators (Fig. 2a and Table 1), which suggested that any interpretation based solely on the correlation between any pair of descriptors has to be evaluated with caution. Complexity, which as explained above, is a rough estimate of how complicated a structure is (Table 1), was significantly and positively correlated with GI_50 _for "all drugs" (0.591; *p *= 2.0 × 10^-2^), intercalators (0.435; *p *= 1.04 × 10^-2^) and 'M-region' compounds (0.984; *p *= 5.23 × 10^-6^). A further analysis of the potential effect of the equilibrium binding constant on the prediction of GI_50 _was undertaken by other approaches described below.

**Figure 4:**
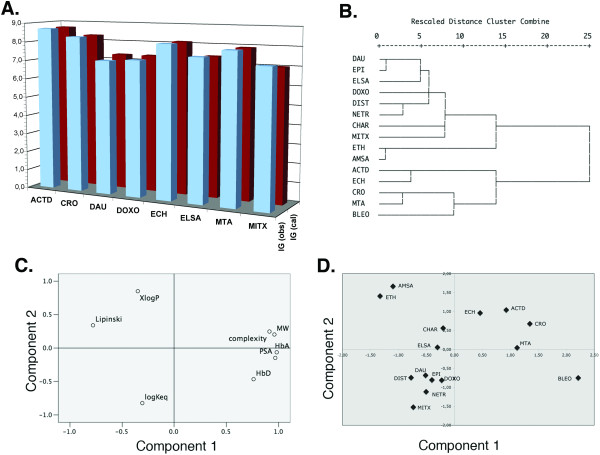
**Molecular descriptors and cell growing inhibition (GI_50_)**. **(A) **Plot comparing the GI_50_, retrieved from the NCI-60 cell lines (Table 1) and the values calculated by multiple regression analysis (GI_50 _(obs)). Equations used to calculate GI_50_are shown in Table 5. The plot corresponds to the more active 'M-region' compounds. **(B) **Dendrograms showing a hierarchical clustering of all DNA-binding drugs, which takes into account all the descriptors, including *logKeq *and Lipinski's scores. **(C) **Principal component analysis of molecular descriptors plus *logKeq *and Lipinski's scores for all the DNA-binding drugs, shown in a rotated space; two-dimensional representation of loading values are shown **(C)**, and the drugs represented according to their factor scores in principal component analysis **(D)**.

Multiple regression was used to derive equations to predict GI_50_, avoiding the problems of multicollinearity. Table 5 shows the equations calculated for either set of drugs ("all" drugs, intercalators and 'M-region' compounds). Statistical details about the multiple regression analysis are shown as supplementary data (Additional File 5), including the VIF (variance inflation factor) values used to detect multicollinearity. Using the *entry *method, see Methods, *logKeq *was included in the predicting equations, taking special care that adding this new variable did not violate the absence of multicollinearity. When the 15 DNA-binding drugs were analyzed together the multiple linear regression analysis excluded *logKeq *as a variable, following the enter/remove criteria outlined in Methods. Much better prediction for GI_50 _was reached in the analysis of the 'intercalators' and 'M-region' subsets, and the predicting equations contained *logKeq *as a variable (Table 5). For the 'M-region' compounds, about 96% of GI_50 _value was predicted multiple regression (Table 5 and Fig. 4A). Unexpectedly, Lipinski's scores (Table 1) did not participate significantly in any of the equations derived to predict GI_50 _values. The main cause for this was multicollinearity.

**Table 5:** Equations used to predict GI_50 _values for DNA-binding drugs^a^.

	**Predictive equation**	**r**	**p**	**AdR^2^**
All drugs^c^	***GI_50 _*= 0.002(± 0.001)Complexity - 0.008(± 0.004)PSA + 5.688(± 0.591)**	0.713	1.4 × 10^-2^	0.426
Intercalators	***GI_50 _*= 0.742(± 0.219)*logKeq *+ 0.002(± 0.000)Complexity + 0.008(± 0.002)PSA + 1.977(± 1.319)**	0.894	1.6 × 10^-2^	0.700
'M-region'	***GI_50 _*= 0.021(± 0.102)*logKeq *+ 0.001(± 0.000)Complexity + 6.478(± 0.746)**	0.984	1.8 × 10^-4^	0.955

The predictive equations are presented for the three sets of drugs analyzed (All 15 drugs, Intercalators and 'M-region' compounds) described in the main text^b^.

^a ^Obtained by multiple regression analysis. The predictive equations displayed are those statistically more significant for each set of predictors (actual *p *values, ANOVA test, are shown in the Table). Other details as in legend to Table 4.

^b^The cases (drugs) used in the calculations for each set were 15, 10 and 8 respectively.

^c^*logKeq *was not a significant predictor for GI_50 _for the set that contains the 15 (all) drugs.

Complexity was the main predictor for cytotoxicity (GI_50 _values, Table 5). This observation was consistent with that more complex molecules would tend to be more potent inhibitors of cell growth [9], while the chance of violating Lipinski's rule is enhanced for large molecules [32]. However, a comparison between Tables 4 and 5 uncovered that complexity was not a relevant variable in the equations aimed at predicting the DNA-binding constant (*logKeq*) described above. Complexity was excluded from the equations used to predict *logKeq*, regardless of its importance in predicting GI_50 _values, because it showed a significant multicollinearity with other parameters related to size. Consistently, complexity loaded close to Mw in the principal component loading plots (Fig. 3).

A hierarchical clustering of all the drugs based on the Pearson correlation coefficients (Fig. 4B) rendered two main clusters that separate the molecules with higher complexity from the rest of compounds. Although at first glance complexity may benefit from a larger size, it also considers other structural features including symmetry (Table 1). Some degree of coherent clustering was expected for molecules related by chemical structure, thus structurally-related molecules clustered nearby, such as the anthracyclines and the intercalator elsamicin A (Figs. 1 and 4B).

Principal component analysis was used to further disclose the relationship between GI_50_, *logKeq *and common molecular descriptors. As seen, the first principal component (Fig. 4C) separated all the size-related descriptors from Lipinski's scores, which evaluates large molecules negatively [32]. The principal component analysis of the more biologically-active 'M-region' compounds produced a correlation matrix that was not positive definite, together with a low extraction of Lipinski's score (46% only; see Additional File 6), thus an additional principal components analysis was performed in which Lipinski's scores were not considered. While the reasons that could render a matrix that was not positively definite are diverse and difficult to delineate, removing Lipinski's score in the analysis was enough to render a meaningful correlation matrix, perhaps indicating that this predictor was essentially not independent of the other molecular descriptors. The extraction of two components using principal component analysis explained about 87%, 88% and 94% of the total variance for all the drugs, intercalators and 'M-region' compounds respectively (Additional File 6). It is noteworthy that the anthracyclines, which are a well referenced group of active antitumor drugs [3] clustered near the negative region of the second principal component in the region of *logKeq *loading (cf. panels C and D in Fig. 4). The huger molecules scored in the region corresponding to the loadings of complexity and XlogP (up-right quadrant in Fig. 4C), while the smaller ethidium and m-AMSA scored consistently in the plot region with higher XlogP and Lipinski loadings.

## Discussion

There is an increasing interest in interfacing the studies on drug cytotoxicity based on the NCI's tumor screening panels with gene expression databases and the mechanisms of drug action, cell sensitivity and resistance [8,14]. These complementary approaches should provide clues about the mechanisms of some molecules, which ultimately can be developed as antitumor agents [10,14,15]. Drugs binding noncovalently to DNA have been in cancer treatment since the 60's, and a detailed structural and functional data on these molecules is available [4], including quantitative data on their binding to DNA as well as the GI_50 _determined in the NCI-60 cell lines. These data sources evidence the selectivity and relative effectiveness of such drugs as anti-cancer agents [1,8,14], which in a few cases has brought about the development of new clinically useful derivatives, such as the anthracycline epirubicin [3].

We can intuitively consider that a certain relationship should exist between the affinity of some drugs for binding to certain DNA sequences and the mechanisms of action, including their cytotoxicity. Hence, it seems possible to facilitate the development of new drugs through a better knowledge of the molecular descriptors that may participate in the strength of binding to DNA. It is anticipated that any prediction of biological activity using physicochemical descriptors is open to some margin of error because there are other aspects that participate in cytotoxicity, such as pharmacokinetics (for example, whether the drugs can easily cross the cell membrane, or overtake a multidrug resistance phenotype). The relation between the capacity of a certain drugs for binding DNA and their biological activity is made evident by the correlation found between physicochemical and biological data for some m-AMSA and actynomycin D analogs, which have been developed from QSAR studies [18,33], and also because DNA-binding drugs may act considered to act by altering gene transcription through the inhibition of the interactions between DNA and certain transcription factors [1,34,35]. Besides some o the drugs shown in Table 1 are also regarded as topoisomerase II poisons, seemingly as a results of their interference with the DNA binding sites for the enzyme, an aspect that is only indirectly addressed here in terms of the drug's equilibrium binding constants. Nevertheless, this makes an interesting point, because it may, for example, explain why m-AMSA is much potent antitumor agent than ethidium (cf. their GI_50 _values in Table 1) even though most of their molecular descriptors are very similar (Table 1). Moreover, m-AMSA is active while o-AMSA, which contains a methoxy group in the *ortho *rather than in the *meta *position, is not [36] regardless of the higher DNA-binding constant of o-AMSA [24]. The m-AMSA activity is explained by its direct interaction with topoisomerase II [36]. In spite of this, the replacement of the acridine moiety with the analogous 2-oxo-2H-pyrano [2,3-b]quinoline system drastically reduced both the anti-cancer activity and the intercalation into DNA [37] in line with the correlation observed for intercalators between *logKeq *and GI_50_.

The analysis presented here represents the first attempt at establishing the bases for a deeper understanding of the links that appear to exist between antitumor activity and drug binding to DNA by evaluating whether molecular descriptors can be used to define noncovalent drug-DNA interactions. A clear correlation between *logKeq *and several molecular descriptors is evident (Table 3). Among them, only XlogP is clearly and 'independent' descriptor, mostly because it is the unique descriptor in Table 1 for which the molecular size is barely relevant (Figs. 3 and 4).

Although, the multiple linear regression method used here cannot capture nonlinear aspects of the relation between *logKeq *or GI_50 _and the molecular descriptors, the approach used in this paper may be replaced by any chosen nonlinear mathematical regression. Multicollinearity has been considered scrupulously given the small number of both drugs and molecular descriptors, thus its occurrence was used to eliminate redundancy in equations aimed at predicting *logKeq *(Table 4) or GI_50 _(Table 5). The computations presented here disclosed that principal component analysis is a rather strong tool for predicting the presence of redundancy in descriptive elements during drug design, helping to clarify the relative importance of each molecular descriptor (Figs. 3 and 4). In general, it can be considered that large molecules, no matter whether they intercalate or not into DNA, may "benefit" of their complexity to become more cytotoxic (Table 1), while several smaller intercalators, such as the anthracyclines, which bind tightly to DNA [16,30], are known useful antitumor agents, tentatively on account of their effects on gene transcription [13,38]. The loadings of the different molecules in the principal component analysis (Fig. 4C) suggest that diverse molecular descriptors, chiefly *Keq *and complexity (Table 5), would participate in the final cytotoxic potential and its predictability.

Lipinski's scores have been widely used as a predictor for bioavailability [32]. The Lipinski's rule-of-five states that small hydrophobic molecules should be better therapeutic agents. The results presented here challenge this view in agreement with reports indicating that Lipinski's scores are not an appropriate molecular descriptor when dealing with large natural products [39], as it is the case with most of the drugs analyzed here. A large set of the molecules shown in Table 1 violates several of the Lipinski's rules, such as having molecular weights over 500 g.mol^-1^, or hydrogen bond donor counts (HbD) higher than five.

In absence of Lipinski's scores in the equations to predict GI_50 _(Table 5), complexity, a measure of how complicated a molecule is (Table 1), emerged as a fundamental predictor for biological activity, in keeping with the view that more complex molecules tend to be more potent antitumor agents [2,9,16]. Among the more potent drugs analyzed here, those with GI_50 _≥ 7, only mitoxantrone has a Lipinski's score higher than 2 (Table 1).

Equations obtained by multiple regression were significantly better at predicting *logKeq *or GI_50 _for the M-region compounds (actinomycin D, chromomycin, daunorubicin, doxorubicin, echinomycin, elsamicin A, mithramycin A and mitoxantrone) than for the subset of intercalators or for the entire set of DNA-binding drugs. About 90% of the experimental *logKeq *and 95% GI_50 _were simulated even after correcting the results for the small sample population (AdR^2 ^values shown in Tables 3 and 4). In SOM the 'M-region' encompasses potent antitumor compounds [9], some of them are DNA-binding drugs, which act mainly by interfering with DNA synthesis and transcription, but it also contains other drugs of natural origin acting against the mitotic spindle, such as taxanes [9].

A potential concern about the approach presented here was to establish the robustness of the statistical tests that, of necessity, were employing a small number of cases (drugs), which represent only about twice the number of variables (molecular descriptors). However, by using both parametric and nonparametric statistic approaches, it has been possible to evaluate the strength of noncovalent drug binding to DNA from common molecular descriptors, and to established whether these molecular characteristics are correlated with their cytotoxic/cytostatic activity in cells in culture. In addition, it will be opportune to link the predictions presented here with the analysis of changes in gene expression induced by those drugs since this may retrieve genes that can be used as predictors of chemosensitivity [8,11,13,40].

Target-specific drugs that bind reversibly to certain DNA sequences with high affinity have been of outstanding interest in the development of new antitumor agents [16,17,41]. A main conclusion of the present study is that both the strength of binding to DNA and drug cytotoxicity are fairly predictable from molecular descriptors, in agreement with that compounds active across the NI-60 cell lines tend to have common structural features [42].

## Conclusion

For drugs binding reversibly to DNA, both their strength of binding and their cytoxicity may be predicted from molecular descriptors by using multiple regression methods. Equations to predict drug-DNA binding constants and growth-inhibitory concentrations were obtained by multiple regression following rigorous statistical procedures. These equations may be useful for rational drug design The results obtained agree with that compounds more active across de National Cancer Institute's 60 cell-line data set tend to have common structural features.

## Supplementary Material

Additional file 1 Pearson correlations (r) and significance levels (*p*-values) calculated between all molecular descriptors and GI_50 _values. Click here for file

Additional file 2 Spearman's ρ correlations and significance levels (*p*-values) calculated between all molecular descriptors and GI_50 _values. Click here for file

Additional file 3 **Data outputs obtained in the multiple regression analyses to predict *logKeq *using molecular descriptors**. Data are presented for the three subsets of drugs: DNA-binding (all) drugs, intercalators, and 'M-region' compounds. Equations used to predict *logKeq *are shown in Table 4. Click here for file

Additional file 4 **Data ouputs generated by the principal component analyses of the molecular descriptors used to predict *logKeq***. Data are presented for the three subsets of drugs: DNA-binding (all) drugs, intercalators and 'M-region' compounds. Click here for file

Additional file 5 **Data outputs obtained in the multiple regression analyses to predict cytotoxicity (GI_50_) using molecular descriptors and the drug-DNA equilibrium binding constant (*logKeq*)**. Data are presented for the three subsets of drugs: DNA-binding (all) drugs, intercalators, and 'M-region' compounds. Equations used to predict GI_50 _are shown in Table 5. Click here for file

Additional file 6 **Data outputs generated by the principal component analyses of the molecular descriptors and the drug-DNA equilibrium binding constant (*logKeq*) used to predict cytotoxicity (GI_50_)**. Data are presented for the three subsets of drugs: DNA-binding (all) drugs, intercalators and 'M-region' compounds. Click here for file
