# Supplementary material for: Evaluation of molecular descriptors for antitumor drugs with respect to noncovalent binding to DNA and antiproliferative activity
Source: BMC Pharmacol. 2009 Sep 16;9:11. doi: 10.1186/1471-2210-9-11 (PMC2758867; doi:10.1186/1471-2210-9-11)
Supplement: Additional file 1 — Pearson correlations (r) and significance levels (p-values) calculated between all molecular descriptors and GI50 values. [file 1471-2210-9-11-S1.pdf]

Additional File 1. Pearson correlations (r) and significance levels (*p*-values) calculated between all molecular descriptors and GI_{50} values[a].

		MW	XlogP	HbD	HbA	PSA	Complexity	*logKeq*	Lipinski	GI_{50}
MW	Pearson Correlation	1	-0.154	.603(**)	.912(**)	.908(**)	.976(**)	-0.396	-.646(**)	.542(*)
	Sig. (1-tailed)		0.292	0.009	0.000	0.000	0.000	0.072	0.005	0.019
	N	15	15	15	15	15	15	15	15	15
XlogP	Pearson Correlation	-0.154	1	-.660(**)	-0.395	-0.434	-0.106	-.461(*)	.505(*)	0.049
	Sig. (1-tailed)	0.292		0.004	0.072	0.053	0.354	0.042	0.028	0.432
	N	15	15	15	15	15	15	15	15	15
HbD	Pearson Correlation	.603(**)	-.660(**)	1	.782(**)	.858(**)	.472(*)	0.047	-.604(**)	-0.023
	Sig. (1-tailed)	0.009	0.004		0.000	0.000	0.038	0.434	0.009	0.468
	N	15	15	15	15	15	15	15	15	15
HbA	Pearson Correlation	.912(**)	-0.395	.782(**)	1	.940(**)	.852(**)	-0.272	-.810(**)	0.439
	Sig. (1-tailed)	0.000	0.072	0.000		0.000	0.000	0.163	0.000	0.051
	N	15	15	15	15	15	15	15	15	15
PSA	Pearson Correlation	.908(**)	-0.434	.858(**)	.940(**)	1	.845(**)	-0.151	-.738(**)	0.286
	Sig. (1-tailed)	0.000	0.053	0.000	0.000		0.000	0.296	0.001	0.151
	N	15	15	15	15	15	15	15	15	15
Complexity	Pearson Correlation	.976(**)	-0.106	.472(*)	.852(**)	.845(**)	1	-0.373	-.627(**)	.591(*)
	Sig. (1-tailed)	0.000	0.354	0.038	0.000	0.000		0.086	0.006	0.010
	N	15	15	15	15	15	15	15	15	15
logKeq	Pearson Correlation	-0.396	-.461(*)	0.047	-0.272	-0.151	-0.373	1	-0.071	-0.277
	Sig. (1-tailed)	0.072	0.042	0.434	0.163	0.296	0.086		0.401	0.159
	N	15	15	15	15	15	15	15	15	15
Lipinski	Pearson Correlation	-.646(**)	.505(*)	-.604(**)	-.810(**)	-.738(**)	-.627(**)	-0.071	1	-0.397
	Sig. (1-tailed)	0.005	0.028	0.009	0.000	0.001	0.006	0.401		0.071
	N	15	15	15	15	15	15	15	15	15
GI_{50}	Pearson Correlation	.542(*)	0.049	-0.023	0.439	0.286	.591(*)	-0.277	-0.397	1
	Sig. (1-tailed)	0.019	0.432	0.468	0.051	0.151	0.010	0.159	0.071	
	N	15	15	15	15	15	15	15	15	15

[a]Correlations were significant at the $*p < 0.05$ or $**p < 0.01$ levels.
