# Supplementary material for: Evaluation of molecular descriptors for antitumor drugs with respect to noncovalent binding to DNA and antiproliferative activity
Source: BMC Pharmacol. 2009 Sep 16;9:11. doi: 10.1186/1471-2210-9-11 (PMC2758867; doi:10.1186/1471-2210-9-11)
Supplement: Additional file 2 — Spearman's ρ correlations and significance levels (p-values) calculated between all molecular descriptors and GI50 values. [file 1471-2210-9-11-S2.pdf]

Additional File 2. Spearman's ρ correlations and significance levels (p-values) calculated between all molecular descriptors and GI_{50} values[a].

Spearman's ρ		MW	XlogP	HbD	HbA	PSA	Complexity	*logKeq*	Lipinski	GI_{50}
MW	Correlation Coefficient	1.000	-0.211	0.361	.969(**)	.864(**)	.983(**)	-0.177	-.747(**)	.635(**)
	Sig. (1-tailed)	.	0.225	0.093	0.000	0.000	0.000	0.264	0.001	0.006
	N	15	15	15	15	15	15	15	15	15
XlogP	Correlation Coefficient	-0.211	1.000	-.883(**)	-0.308	-.452(*)	-0.131	-0.388	.502(*)	-0.042
	Sig. (1-tailed)	0.225	.	0.000	0.132	0.045	0.321	0.077	0.028	0.441
	N	15	15	15	15	15	15	15	15	15
HbD	Correlation Coefficient	0.361	-.883(**)	1.000	.475(*)	.573(*)	0.281	0.252	-.624(**)	0.057
	Sig. (1-tailed)	0.093	0.000	.	0.037	0.013	0.155	0.183	0.006	0.420
	N	15	15	15	15	15	15	15	15	15
HbA	Correlation Coefficient	.969(**)	-0.308	.475(*)	1.000	.911(**)	.950(**)	-0.244	-.837(**)	.618(**)
	Sig. (1-tailed)	0.000	0.132	0.037	.	0.000	0.000	0.191	0.000	0.007
	N	15	15	15	15	15	15	15	15	15
PSA	Correlation Coefficient	.864(**)	-.452(*)	.573(*)	.911(**)	1.000	.875(**)	-0.211	-.857(**)	.486(*)
	Sig. (1-tailed)	0.000	0.045	0.013	0.000	.	0.000	0.225	0.000	0.033
	N	15	15	15	15	15	15	15	15	15
Complexity	Correlation Coefficient	.983(**)	-0.131	0.281	.950(**)	.875(**)	1.000	-0.252	-.735(**)	.632(**)
	Sig. (1-tailed)	0.000	0.321	0.155	0.000	0.000	.	0.182	0.001	0.006
	N	15	15	15	15	15	15	15	15	15
logKeq	Correlation Coefficient	-0.177	-0.388	0.252	-0.244	-0.211	-0.252	1.000	0.114	-0.256
	Sig. (1-tailed)	0.264	0.077	0.183	0.191	0.225	0.182	.	0.343	0.179
	N	15	15	15	15	15	15	15	15	15
Lipinski	Correlation Coefficient	-.747(**)	.502(*)	-.624(**)	-.837(**)	-.857(**)	-.735(**)	0.114	1.000	-0.429
	Sig. (1-tailed)	0.001	0.028	0.006	0.000	0.000	0.001	0.343	.	0.055
	N	15	15	15	15	15	15	15	15	15
GI_{50}	Correlation Coefficient	.635(**)	-0.042	0.057	.618(**)	.486(*)	.632(**)	-0.256	-0.429	1.000
	Sig. (1-tailed)	0.006	0.441	0.420	0.007	0.033	0.006	0.179	0.055	.
	N	15	15	15	15	15	15	15	15	15

a. Correlations were significant at the $*p < 0.05$ or $**p < 0.01$ levels.
