# Supplementary material for: Evaluation of molecular descriptors for antitumor drugs with respect to noncovalent binding to DNA and antiproliferative activity
Source: BMC Pharmacol. 2009 Sep 16;9:11. doi: 10.1186/1471-2210-9-11 (PMC2758867; doi:10.1186/1471-2210-9-11)
Supplement: Additional file 3 — Data outputs obtained in the multiple regression analyses to predict logKeq using molecular descriptors. Data are presented for the three subsets of drugs: DNA-binding (all) drugs, intercalators, and 'M-region' compounds. Equations used to predict logKeq are shown in Table 4. [file 1471-2210-9-11-S3.pdf]

Additional File 3. Data outputs obtained in the multiple regression analyses to predict *logKeq* using molecular descriptors. Data are presented for the three subsets of drugs: DNA-binding (all) drugs, intercalators, and 'M-region' compounds. Equations used to predict *logKeq* are shown in Table 4.

DNA-binding drugs (model 1)

Variables Entered/Removed(b)

Model	Variables Entered	Variables Removed	Method
1	PSA. XlogP(a)	.	Enter

a. All requested variables entered.
b. Dependent Variable: logKeq

Model Summary(b)

Model	R	R Square	Adjusted R Square	Std. Error of the Estimate
1	.603(a)	0.364	0.258	0.74819

a. Predictors: (Constant). PSArea. XlogP
b. Dependent Variable: logKeq

ANOVA(b)

Model		Sum of Squares	df	Mean Square	F	Sig.
1	Regression	3.840	2	1.920	3.430	6.60.E-02 (a)
	Residual	6.717	12	0.560		
	Total	10.558	14			

a. Predictors: (Constant). PSArea. XlogP
b. Dependent Variable: logKeq

Coefficients

Model		Unstandardized Coefficients		Standardized Coefficients	t	Sig.	Collinearity Statistics	
		B	Std. Error	Beta			Tolerance	VIF
1	(Constant)	6.603	0.465		14.200	7.26E-09		
	XlogP	-0.255	0.100	-0.648	-2.536	0.026	0.812	1.232
	PSA	-0.003	0.002	-0.432	-1.690	0.117	0.812	1.232

DNA-binding drugs (model 2)

Variables Entered/Removed(b)

Model	Variables Entered	Variables Removed	Method
2	XlogP(a)	.	Enter

a. All requested variables entered.
b. Dependent Variable: logKeq

Model Summary(b)

Model	R	R Square	Adjusted R Square	Std. Error of the Estimate
2	.461(a)	0.212	0.152	0.79981

a. Predictors: (Constant). XlogP
b. Dependent Variable: logKeq

ANOVA(b)

Model		Sum of Squares	df	Mean Square	F	Sig.
2	Regression	2.242	1	2.242	3.504	8.40.E-02 (a)
	Residual	8.316	13	0.640		
	Total	10.558	14			

a. Predictors: (Constant). XlogP
b. Dependent Variable: logKeq

Coefficients

Model		Unstandardized Coefficients		Standardized Coefficients	t	Sig.	Correlations			Collinearity Statistics	
			Std. Error	Beta			Zero-order	Partial	Part	Tolerance	VIF
2	(Constant)	5.895	0.215		27.412	6.9.E-13					
	XlogP	-0.181	0.097	-0.461	-1.872	0.084	-0.461	-0.461	-0.461	1.000	1.000

Intercalators

Variables Entered/Removed(a)

Model	Variables Entered	Variables Removed	Method
	XlogP	.	Stepwise (Criteria: Probability-of-F-to-enter <= .050. Probability-of-F-to-remove >= .100).

a. Dependent Variable: logKeq

Model Summary(b)

Model	R	R Square	Adjusted R Square	Std. Error of the Estimate
	.662(a)	0.438	0.368	0.67388

a. Predictors: (Constant). XlogP
b. Dependent Variable: logKeq

ANOVA(b)

Model	Sum of Squares	df	Mean Square	F	Sig.
Regression	2.833	1	2.833	6.238	3.70.E-02 (a)
Residual	3.633	8	0.454		
Total	6.466	9			

a. Predictors: (Constant). XlogP
b. Dependent Variable: logKeq

Coefficients

Model		Unstandardized Coefficients		Standardized Coefficients	t	Sig.	Correlations			Collinearity Statistics	
		B	Std. Error	Beta			Zero-order	Partial	Part	Tolerance	VIF
	(Constant)	6.054	0.229		26.431	4.51E-09					
	XlogP	-0.225	0.090	-0.662	-2.498	0.037	-0.662	-0.662	-0.662	1.000	1.000

<u>'M-region' compounds (model 1)</u>

Variables Entered/Removed(b)

Model	Variables Entered	Variables Removed	Method
1	XlogP. HbA. HbD(a)	.	Enter

a. All requested variables entered.
b. Dependent Variable: logKeq

Model Summary(b)

Model	R	R Square	Adjusted R Square	Std. Error of the Estimate
1	.984(a)	0.968	0.944	0.20411

a. Predictors: (Constant). XlogP. HbA. HbD
b. Dependent Variable: logKeq

ANOVA(b)

Model		Sum of Squares	df	Mean Square	F	Sig.
1	Regression	5.011	3	1.670	40.093	2.00.E-03 (a)
	Residual	0.167	4	0.042		
	Total	5.177	7			

a. Predictors: (Constant). XlogP. HbA. HbD
b. Dependent Variable: logKeq

Coefficients

Model		Unstandardized Coefficients		Standardized Coefficients	t	Sig.	Collinearity Statistics	
		B	Std. Error	Beta			Tolerance	VIF
1	(Constant)	7.577	0.298		25.438	0.000		
	HbD	0.173	0.066	0.470	2.614	0.059	0.249	4.011
	HbA	-0.178	0.020	-1.230	-8.822	0.001	0.414	2.417
	XlogP	0.128	0.067	0.291	1.904	0.130	0.344	2.905

‘M-region’ compounds (model 2)

Variables Entered/Removed(a)

Model	Variables Entered	Variables Removed	Method
2	HbA	.	Stepwise (Criteria: Probability-of-F-to-enter <= .050 of-F-to-remove >= .100).

a. Dependent Variable: logKeq

Model Summary(b)

Model	R	R Square	Adjusted R Square	Std. Error of the Estimate
2	.955(a)	0.912	0.897	0.27587

a. Predictors: (Constant). HbA
b. Dependent Variable: logKeq

ANOVA(b)

Model		Sum of Squares	df	Mean Square	F	Sig.
2	Regression	4.721	1	4.721	62.032	2.20E-4 (a)
	Residual	0.457	6	0.076		
	Total	5.177	7			

a. Predictors: (Constant). HbA

b. Dependent Variable: logKeq

Coefficients

Model		Unstandardized Coefficients		Standardized Coefficients	t	Sig.	Collinearity Statistics	
		B	Std. Error	Beta			Tolerance	VIF
2	(Constant)	8.092	0.303		26.703	0.000		
	HbA	-0.138	0.018	-0.955	-7.876	0.000	1.000	1.000
