# Supplementary material for: Evaluation of molecular descriptors for antitumor drugs with respect to noncovalent binding to DNA and antiproliferative activity
Source: BMC Pharmacol. 2009 Sep 16;9:11. doi: 10.1186/1471-2210-9-11 (PMC2758867; doi:10.1186/1471-2210-9-11)
Supplement: Additional file 4 — Data ouputs generated by the principal component analyses of the molecular descriptors used to predict logKeq. Data are presented for the three subsets of drugs: DNA-binding (all) drugs, intercalators and 'M-region' compounds. [file 1471-2210-9-11-S4.pdf]

Additional File 4. Data ouputs generated by the principal component analyses of the molecular descriptors used to predict *logKeq*. Data are presented for the three subsets of drugs: DNA-binding (all) drugs, intercalators and M-region compounds.

DNA-binding drugs

Communalities

	Initial	Extraction
MW	1.000	.989
XlogP	1.000	.907
HbD	1.000	.894
HbA	1.000	.946
PSA	1.000	.975
complexity	1.000	.949

Extraction Method: Principal Component Analysis.

Total Variance Explained

Component	Initial Eigenvalues			Extraction Sums of Squared Loadings			Rotation Sums of Squared Loadings		
	Total	% of Variance	Cumulative %	Total	% of Variance	Cumulative %	Total	% of Variance	Cumulative %
1	4.453	74.222	74.222	4.453	74.222	74.222	3.741	62.343	62.343
2	1.208	20.132	94.354	1.208	20.132	94.354	1.921	32.010	94.354
3	.251	4.182	98.536						
4	.070	1.160	99.695						
5	.014	.227	99.922						
6	.005	.078	100.000						

Extraction Method: Principal Component Analysis.

Component Matrix(a)

	Component	
	1	2
MW	.929	.354
XlogP	-.463	.832
HbD	.835	-.444
HbA	.971	.055
PSA	.988	-.006
complexity	.872	.435

Extraction Method: Principal Component Analysis.
a 2 components extracted.

Rotated Component Matrix(a)

	Component	
	1	2
MW	.987	.122
XlogP	-.019	-.952
HbD	.529	.784
HbA	.884	.406
PSA	.870	.468
complexity	.974	.025

Extraction Method: Principal Component Analysis. Rotation Method: Varimax with Kaiser Normalization.
a Rotation converged in 3 iterations.

Component Transformation Matrix

Component	1	2
1	.883	.469
2	.469	-.883

Extraction Method: Principal Component Analysis.
Rotation Method: Varimax with Kaiser Normalizatio

Intercalators

Communalities

	Initial	Extraction
MW	1.000	.989
XlogP	1.000	.919
HbD	1.000	.909
HbA	1.000	.963
PSA	1.000	.990
complexity	1.000	.934

Extraction Method: Principal Component Analysis.

Total Variance Explained

Component	Initial Eigenvalues			Extraction Sums of Squared Loadings			Rotation Sums of Squared Loadings		
	Total	% of Variance	Cumulative %	Total	% of Variance	Cumulative %	Total	% of Variance	Cumulative %
1	4.664	77.740	77.740	4.664	77.740	77.740	3.788	63.125	63.125
2	1.041	17.347	95.087	1.041	17.347	95.087	1.918	31.961	95.087
3	.251	4.180	99.266						
4	.040	.671	99.937						
5	.004	.059	99.995						
6	.000	.005	100.000						

Extraction Method: Principal Component Analysis.

Component Matrix(a)

	Component	
	1	2
MW	.940	.324
XlogP	-.537	.794
HbD	.891	-.340
HbA	.981	.027
PSA	.995	.024
complexity	.864	.434

Extraction Method: Principal Component Analysis.
a 2 components extracted.

Rotated Component Matrix(a)

	Component	
	1	2
MW	.978	.181
XlogP	-.077	-.956
HbD	.608	.734
HbA	.867	.460
PSA	.878	.468
complexity	.965	.047

Extraction Method: Principal Component Analysis.
Rotation Method: Varimax with Kaiser Normalization.
a Rotation converged in 3 iterations.

Component Transformation Matrix

Component	1	2
1	.871	.492
2	.492	-.871

Extraction Method: Principal Component Analysis.
Rotation Method: Varimax with Kaiser Normalization.

‘M-region’ compounds

Communalities

	Initial	Extraction
MW	1.000	.981
XlogP	1.000	.855
HbD	1.000	.933
HbA	1.000	.929
PSA	1.000	.991
complexity	1.000	.948

Extraction Method: Principal Component Analysis.

Total Variance Explained

Component	Initial Eigenvalues			Extraction Sums of Squared Loadings			Rotation Sums of Squared Loadings		
	Total	% of Variance	Cumulative %	Total	% of Variance	Cumulative %	Total	% of Variance	Cumulative %
1	3.850	64.164	64.164	3.850	64.164	64.164	3.847	64.109	64.109
2	1.789	29.814	93.978	1.789	29.814	93.978	1.792	29.869	93.978
3	.281	4.691	98.669						
4	.063	1.056	99.725						
5	.010	.170	99.895						
6	.006	.105	100.000						

Extraction Method: Principal Component Analysis.

Component Matrix(a)

	Component	
	1	2
MW	.986	-.091
XlogP	.352	-.855
HbD	.223	.940
HbA	.905	.333
PSA	.992	.086
complexity	.949	-.217

Extraction Method: Principal Component Analysis.
a 2 components extracted.

Rotated Component Matrix(a)

	Component	
	1	2
MW	.982	-.130
XlogP	.317	-.869
HbD	.261	.930
HbA	.917	.297
PSA	.995	.046
complexity	.940	-.255

Extraction Method: Principal Component Analysis.
Rotation Method: Varimax with Kaiser Normalization.
a Rotation converged in 3 iterations.

Component Transformation Matrix

Component	1	2
1	.999	-.040
2	.040	.999

Extraction Method: Principal Component Analysis.
Rotation Method: Varimax with Kaiser Normalization.
