# Supplementary material for: Evaluation of molecular descriptors for antitumor drugs with respect to noncovalent binding to DNA and antiproliferative activity
Source: BMC Pharmacol. 2009 Sep 16;9:11. doi: 10.1186/1471-2210-9-11 (PMC2758867; doi:10.1186/1471-2210-9-11)
Supplement: Additional file 5 — Data outputs obtained in the multiple regression analyses to predict cytotoxicity (GI50) using molecular descriptors and the drug-DNA equilibrium binding constant (logKeq). Data are presented for the three subsets of drugs: DNA-binding (all) drugs, intercalators, and 'M-region' compounds. Equations used to predict GI50 are shown in Table 5. [file 1471-2210-9-11-S5.pdf]

Additional File 5. Data outputs obtained in the multiple regression analyses to predict cytotoxicity (GI_{50}) using structure-based molecular descriptors and the drug-DNA equilibrium binding constant (*logKeq*). Data are presented for the three subsets of drugs: DNA-binding (all) drugs, intercalators, and 'M-region' compounds. Equations used to predict GI_{50} are shown in Table 5.

DNA-binding drugs

Variables Entered/Removed(b)

Model	Variables Entered	Variables Removed	Method
	PSA. complexity(a)	.	Enter

a. All requested variables entered.
b. Dependent Variable: GI50

Model Summary(b)

Model	R	R Square	Adjusted R Square	Std. Error of the Estimate
	.713(a)	0.508	0.426	1.0883

a. Predictors: (Constant). PSA. complexity
b. Dependent Variable: GI50

ANOVA(b)

Model	Sum of Squares	df	Mean Square	F	Sig.
Regression	14.679	2	7.339	6.196	1.4.E-02 (a)
Residual	14.214	12	1.185		
Total	28.893	14			

a. Predictors: (Constant). PSA. complexity
b. Dependent Variable: GI50

Coefficients(a)

Model	Unstandardized Coefficients		Standardized Coefficients	t	Sig.	Collinearity Statistics	
	B	Std. Error	Beta			Tolerance	VIF
(Constant)	5.688	0.591		9.620	0.000		
complexity	0.002	0.001	1.220	3.224	0.007	0.286	3.495
PSA	-0.008	0.004	-0.745	-1.968	0.073	0.286	3.495

Intercalators

Variables Entered/Removed(b)

Model	Variables Entered	Variables Removed	Method
	PSA. logKeq. complexity(a)	.	Enter

a. All requested variables entered.
b. Dependent Variable: GI50

Model Summary(b)

Model	R	R Square	Adjusted R Square	Std. Error of the Estimate
	.894(a)	0.800	0.700	0.5298

a. Predictors: (Constant). PSA. logKeq. complexity
b. Dependent Variable: GI50

ANOVA(b)

Model	Sum of Squares	df	Mean Square	F	Sig.
Regression	6.727	3	2.242	7.990	1.6.E-02 (a)
Residual	1.684	6	0.281		
Total	8.411	9			

a. Predictors: (Constant). PSA. logKeq. complexity
b. Dependent Variable: GI50

Coefficients(a)

Model	Unstandardized Coefficients		Standardized Coefficients	t	Sig.	Collinearity Statistics	
	B	Std. Error	Beta			Tolerance	VIF
(Constant)	1.977	1.319		1.499	0.185		
logKeq	0.742	0.219	0.650	3.391	0.015	0.908	1.102
complexity	0.002	0.000	1.558	4.316	0.005	0.256	3.905
PSA	-0.008	0.002	-1.256	-3.482	0.013	0.256	3.899

‘M-region’ compounds

Variables Entered/Removed(b)

Model	Variables Entered	Variables Removed	Method
	complexity. logKeq(a)	.	Enter

a. All requested variables entered.
b. Dependent Variable: GI50

Model Summary(b)

Model	R	R Square	Adjusted R Square	Std. Error of the Estimate
	.984(a)	0.968	0.955	0.1247

a. Predictors: (Constant). complexity. logKeq
b. Dependent Variable: GI50

ANOVA(b)

Model		Sum of Squares	df	Mean Square	F	Sig.
	Regression	2.362	2	1.181	76.007	1.8.E-04 (a)
	Residual	0.078	5	0.016		
	Total	2.440	7			

a. Predictors: (Constant). complexity. logKeq
b. Dependent Variable: GI50

Coefficients(a)

Model		Unstandardized Coefficients		Standardized Coefficients	t	Sig.	Collinearity Statistics	
		B	Std. Error	Beta			Tolerance	VIF
	(Constant)	6.478	0.746		8.680	0.000		
	logKeq	0.021	0.102	0.031	0.207	0.844	0.287	3.483
	complexity	0.001	0.000	1.010	6.780	0.001	0.287	3.483
