# Supplementary material for: Evaluation of molecular descriptors for antitumor drugs with respect to noncovalent binding to DNA and antiproliferative activity
Source: BMC Pharmacol. 2009 Sep 16;9:11. doi: 10.1186/1471-2210-9-11 (PMC2758867; doi:10.1186/1471-2210-9-11)
Supplement: Additional file 6 — Data outputs generated by the principal component analyses of the molecular descriptors and the drug-DNA equilibrium binding constant (logKeq) used to predict cytotoxicity (GI50). Data are presented for the three subsets of drugs: DNA-binding (all) drugs, intercalators and 'M-region' compounds. [file 1471-2210-9-11-S6.pdf]

Additional File 6. Data ouputs generated by the principal component analyses of the structure-based molecular descriptors and the drug-DNA equilibrium binding const (*logKeq*) used to predict cytotoxicity (GI_{50}). Data are presented for the three subsets of drugs: DNA-binding (all) drugs, intercalators and 'M-region' compounds.

DNA-binding drugs

Communalities

	Initial	Extraction
MW	1.000	.962
XlogP	1.000	.846
HbD	1.000	.795
HbA	1.000	.967
PSA	1.000	.958
complexity	1.000	.893
logKeq	1.000	.773
Lipinski	1.000	.723

Extraction Method: Principal Component Analysis.

Total Variance Explained

Component	Initial Eigenvalues			Extraction Sums of Squared Loadings			Rotation Sums of Squared Loadings		
	Total	% of Variance	Cumulative %	Total	% of Variance	Cumulative %	Total	% of Variance	Cumulative %
1	5.118	63.979	63.979	5.118	63.979	63.979	5.056	63.204	63.204
2	1.799	22.493	86.473	1.799	22.493	86.473	1.861	23.268	86.473
3	.508	6.354	92.827						
4	.321	4.014	96.840						
5	.209	2.610	99.450						
6	.033	.407	99.857						
7	.008	.095	99.952						
8	.004	.048	100.000						

Extraction Method: Principal Component Analysis.

Component Matrix(a)

	Component	
	1	2
MW	.923	.332
XlogP	-.464	.794
HbD	.817	-.357
HbA	.981	.072
PSA	.979	-.014
complexity	.870	.369
logKeq	-.193	-.858
Lipinski	-.819	.231

Extraction Method: Principal Component Analysis.
a 2 components extracted.

Rotated Component Matrix(a)

	Component	
	1	2
MW	.960	.203
XlogP	-.351	.850
HbD	.760	-.466
HbA	.982	-.063
PSA	.967	-.148
complexity	.912	.247
logKeq	-.308	-.823
Lipinski	-.779	.341

Extraction Method: Principal Component Analysis.
Rotation Method: Varimax with Kaiser Normalization.
a Rotation converged in 3 iterations.

Component Transformation Matrix

Component	1	2
1	.991	-.137
2	.137	.991

Extraction Method: Principal Component Analysis.
Rotation Method: Varimax with Kaiser Normalization.

Intercalators

Communalities

	Initial	Extraction
MW	1.000	.972
XlogP	1.000	.819
HbD	1.000	.790
HbA	1.000	.974
PSA	1.000	.986
complexity	1.000	.871
logKeq	1.000	.873
Lipinski	1.000	.738

Extraction Method: Principal Component Analysis.

Total Variance Explained

Component	Initial Eigenvalues			Extraction Sums of Squared Loadings			Rotation Sums of Squared Loadings		
	Total	% of Variance	Cumulative %	Total	% of Variance	Cumulative %	Total	% of Variance	Cumulative %
1	5.267	65.837	65.837	5.267	65.837	65.837	4.749	59.359	59.359
2	1.758	21.972	87.809	1.758	21.972	87.809	2.276	28.450	87.809
3	.578	7.229	95.038						
4	.258	3.222	98.260						
5	.122	1.520	99.780						
6	.015	.191	99.971						
7	.002	.027	99.998						
8	.000	.002	100.000						

Extraction Method: Principal Component Analysis.

Component Matrix(a)

	Component	
	1	2
MW	.903	.395
XlogP	-.593	.684
HbD	.884	-.095
HbA	.981	.105
PSA	.981	.153
complexity	.834	.420
logKeq	.242	-.902
Lipinski	-.799	.316

Extraction Method: Principal Component Analysis.
a 2 components extracted.

Rotated Component Matrix(a)

	Component	
	1	2
MW	.986	-.018
XlogP	-.284	-.859
HbD	.780	.427
HbA	.946	.280
PSA	.965	.236
complexity	.931	-.067
logKeq	-.123	.926
Lipinski	-.616	-.599

Extraction Method: Principal Component Analysis.
Rotation Method: Varimax with Kaiser Normalization.
a Rotation converged in 3 iterations.

Component Transformation Matrix

Component	1	2
1	.923	.384
2	.384	-.923

Extraction Method: Principal Component Analysis.
Rotation Method: Varimax with Kaiser Normalization.

‘M-region’ compounds

Correlation Matrix(a) is not positive definite when Lipinski’s score is introduced

Communalities (with Lipinski’s score)

	Initial	Extraction
MW	1,000	,955
XlogP	1,000	,825
HbD	1,000	,910
HbA	1,000	,955
PSArea	1,000	,976
complexity	1,000	,929
logKeq	1,000	,965
Lipinski	1,000	,464

Extraction Method: Principal Component Analysis.

Communalities (without Lipinski’s score)

	Initial	Extraction
MW	1.000	.978
XlogP	1.000	.857
HbD	1.000	.920
HbA	1.000	.940
PSArea	1.000	.987
complexity	1.000	.945
logKeq	1.000	.958

Extraction Method: Principal Component Analysis.

Total Variance Explained (without Lipinski’s score)

Component	Initial Eigenvalues			Extraction Sums of Squared Loadings			Rotation Sums of Squared Loadings		
	Total	% of Variance	Cumulative %	Total	% of Variance	Cumulative %	Total	% of Variance	Cumulative %
1	4.765	68.071	68.071	4.765	68.071	68.071	4.765	68.069	68.069
2	1.821	26.012	94.083	1.821	26.012	94.083	1.821	26.014	94.083
3	.284	4.060	98.143						
4	.105	1.507	99.650						
5	.017	.237	99.887						
6	.007	.099	99.986						
7	.001	.014	100.000						

Extraction Method: Principal Component Analysis.

Component Matrix(a)

	Component	
	1	2
MW	.976	.159
XlogP	.285	.881
HbD	.279	-.918
HbA	.931	-.271
PSA	.994	-.016
complexity	.930	.283
logKeq	-.967	.151

Extraction Method: Principal Component Analysis.
a 2 components extracted.

Rotated Component Matrix(a)

	Component	
	1	2
MW	.975	.166
XlogP	.279	.882
HbD	.284	-.916
HbA	.932	-.265
PSA	.994	-.010
complexity	.928	.289
logKeq	-.968	.145

Extraction Method: Principal Component Analysis.
Rotation Method: Varimax with Kaiser Normalization.
a Rotation converged in 3 iterations.

Component Transformation Matrix

Component	1	2
1	1.000	.006
2	-.006	1.000

Extraction Method: Principal Component Analysis.
Rotation Method: Varimax with Kaiser Normalization.
